# Special Issue “Understanding Sports-Related Health Issues, 2nd Edition”

**DOI:** 10.3390/jfmk10040386

**Published:** 2025-10-03

**Authors:** Daniel Rojas-Valverde

**Affiliations:** 1 Clínica de Lesiones Deportivas (Rehab & Readapt), Escuela de Ciencias del Movimiento Humano y Calidad de Vida, Universidad Nacional, Heredia 86-3000, Costa Rica; drojasv@una.cr; Tel.: +506-88250219; 2 Centro de Investigación, Desarrollo e Innovación en Salud y Deporte (CIDISAD), Escuela de Ciencias del Movimiento Humano y Calidad de Vida, Universidad Nacional, Heredia 86-3000, Costa Rica

Sports-related health issues represent a complex and multifactorial phenomenon that extends far beyond the immediate occurrence of an injury or the onset of an illness. They must be understood as dynamic processes that continuously interact with physiological, psychological, and environmental factors. Rather than static events, health disturbances in athletes unfold across time, reflecting the ongoing interplay between stressors, resilience, and adaptation. This view moves away from traditional reductionist perspectives and emphasizes a system-oriented understanding where outcomes are shaped not only by the initial event but also by how the body regulates, compensates, and reorganizes through allostasis [1,2].

When an athlete faces a health problem, there is often an acute transitional decline in structural or functional integrity. This may be expressed as reduced physical performance, fatigue, recurrent infections, or disruption of psychological well-being. While this decline is a critical moment, it should not be considered an endpoint. Instead, it marks the beginning of a trajectory where various outcomes are possible and can potentially be developed. Depending on the adequacy of rest, medical treatment, rehabilitation, psychological support, and contextual factors such as workload or social environment, the athlete may follow distinct pathways of adaptation and recovery [3,4].

One of these pathways involves structural and functional improvement. When recovery is adequately guided and supported, the body not only returns to its baseline level but also adapts to the stressor in a way that strengthens resilience. This reflects the principle that health challenges, when appropriately addressed, can create opportunities for growth, enhanced resistance, and better performance sustainability. Another possible trajectory is one of recovery and sustained reversal, where the individual regains health status and stabilizes without necessarily surpassing the pre-illness condition. This path is often observed when athletes comply with rehabilitation and medical guidance, but where adaptation is limited to re-establishing prior equilibrium. Finally, there is the possibility of structural deterioration or long-term decline [5]. In cases where stressors accumulate, medical management is inadequate, or maladaptive behaviors persist, health disturbances may evolve into chronic problems that compromise long-term well-being and athletic career longevity.

This conceptualization aligns with recent discussions in the scientific literature, where illness and health issues in sport are viewed not simply as irreversible or unidirectional setbacks, but as reversible involutional processes. In this framework, decline is temporary and can be positively influenced by interventions that address not only the physical symptoms but also the broader psychosocial and behavioral context. A recently published framework emphasizes the importance of perceiving setbacks as dynamic and modifiable conditions [6]. Extending this notion to health issues such as overtraining syndrome, recurrent respiratory illnesses, or stress-related disorders allows us to reframe the athlete’s experience of illness from a deterministic decline toward a reversible state that can be redirected with appropriate care [7,8,9].

Central to this discussion is the concept of allostasis, which describes the process by which the body maintains stability through change. Unlike homeostasis, which emphasizes the maintenance of a fixed internal state, allostasis recognizes that health depends on the body’s ability to regulate in response to challenges adaptively. Repeated exposure to stress, whether physiological or psychological, creates an allostatic load. If this load is balanced by sufficient recovery and adaptive capacity, health improves or stabilizes [1,4,10]. However, if the load is excessive or sustained without recovery, maladaptive consequences such as immune dysfunction, chronic fatigue, or structural deterioration may occur. Thus, sports-related health issues can be understood as outcomes of how effectively the body manages allostatic demands over time [11,12].

Adopting this perspective also underscores the importance of holistic, multidimensional interventions. Medical care should not be isolated from psychological support, training adjustments, or lifestyle modifications. Instead, a comprehensive strategy that integrates physical rehabilitation, nutrition, sleep optimization, mental health care, and contextual support from coaches and families is essential [1,6,13]. This integrative approach ensures that athletes not only recover from acute health disturbances but also develop the resilience needed to prevent recurrence and support long-term well-being.

Finally, understanding sports-related health issues through this lens emphasizes that illness is neither inevitable nor irreversible. By acknowledging the dynamic interplay of stress, adaptation, and recovery, and by implementing multidimensional strategies, it is possible to transform setbacks into opportunities for growth [14,15] (see Figure 1). This paradigm not only benefits athletic performance but also protects the holistic health of athletes throughout their careers and beyond their sporting life. Figure 1 illustrates how athletes move from an initial baseline state through cycles of acute loads or stressors that disrupt homeostasis. With appropriate recovery, athletes maintain an allostatic balance, reflecting stability through change. However, repeated stress events can accumulate, producing a transitional functional decline that may lead to an illness or health disturbance.

The Special Issue “Understanding Sports-Related Health Issues, 2nd Edition” aimed to advance knowledge on the multifaceted challenges athletes face regarding health, performance, and well-being. By gathering empirical studies, systematic reviews, and applied analyses, the Special issue sought to integrate perspectives from injury epidemiology, technology and biomarkers, biomechanical risks, and broader health-related outcomes across diverse sporting contexts.

From an epidemiological perspective, training at altitude during the Portuguese Rugby XV World Cup preparation highlighted a dual burden of increased injury incidence and altered performance metrics (Contributor 1). Similarly, a survey of Brazilian Jiu-Jitsu athletes in Italy demonstrated the high frequency of traumatic injuries in combat sports and the lack of standardized rehabilitation pathways (Contributor 2). These findings suggest the urgent need for structured monitoring and return-to-sport protocols.

In the area of technology and physiological monitoring, one study evaluated the accuracy of the Apple Watch Series 9 across athletes with different skin pigmentations, revealing moderate reliability but discrepancies that raise concerns about inclusivity and fairness (Contributor 3). Complementarily, a 10-day multi-stressor field training program demonstrated significant oxidative stress responses in the glutathione system, showing how combined environmental, physical, and psychological stressors challenge resilience (Contributor 4). Both contributions underscore the promise of new monitoring strategies while also pointing to the limitations of current tools.

Addressing biomechanical and sport-specific risks, a systematic review on soccer ball characteristics showed that factors such as inflation, mass, and stiffness strongly modulate head impact forces (Contributor 5). However, the predominance of mechanical models over live-athlete studies limits ecological validity. Adding a sociocultural dimension, an investigation into women’s rugby revealed widespread underreporting of concussions due to stigma, lack of awareness, and competitive pressures (Contributor 6). These findings highlight the need for both equipment modifications and educational interventions that are sensitive to gender and context.

Turning to broader health outcomes, a meta-analysis confirmed that physical exercise is effective in improving multiple indicators of metabolic syndrome, including blood pressure, lipid profiles, and abdominal circumference (Contributor 7). Extending the lifespan perspective, a study of elderly women engaged in lifelong team handball or football demonstrated better bone health and body composition compared to sedentary peers (Contributor 8). Finally, a preliminary evaluation among collegiate female runners suggested that self-reported training volume may serve as an adjunct measure for identifying the risk of the Female Athlete Triad (Contributor 9). These contributions illustrate both the protective role of sustained sport participation and the importance of early detection of sport-specific health risks.

Sports-related health issues are dynamic processes shaped by physiological, psychological, and environmental factors, best understood through the lens of allostasis. Depending on recovery and support, athletes may improve resilience, return to baseline, or suffer long-term decline.

The contributions in this Special Issue highlight the need for integrative frameworks that combine monitoring, technology, epidemiology, biomechanics, and sociocultural awareness. Health challenges should be seen as modifiable trajectories, requiring holistic strategies (medical, psychological, and contextual) that ensure recovery, resilience, and sustainable athlete well-being.

## Figures and Tables

**Figure 1:**
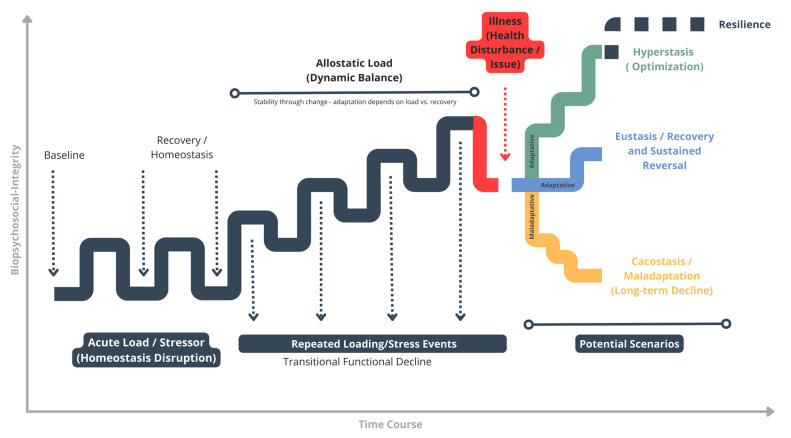
Conceptual model of the dynamic pathways of sports-related health issues.
